# Detection of hepatitis E virus RNA from pig bile collected at a slaughterhouse in Japan

**DOI:** 10.3934/microbiol.2022036

**Published:** 2022-12-26

**Authors:** Masashi Uema, Kenzo Yonemitsu, Yoshimasa Sasaki, Hiroshi Asakura

**Affiliations:** 1 National Institute of Health Sciences, 3-25-26, Tonomachi, Kawasaki-ku, Kawasaki, Kanagawa 210-9501, Japan; 2 Murayama Brunch, National Institute of Infectious Diseases, 4-7-1, Gakuen, Musashimurayama, Tokyo 208-0011, Japan

**Keywords:** hepatitis E virus, pig, bile, liver, slaughterhouse

## Abstract

Hepatitis E virus (HEV) is a zoonotic pathogen that circulates mainly between pigs and humans. In Japan, the number of confirmed HEV cases has increased over the past decade, with the majority reported as domestic HEV infections. HEV-infected pork products may be associated with this increase, but there is limited information on HEV in pork in Japanese markets.

From February to March 2020, gallbladders were collected from 200 slaughtered pigs shipped from 14 farms and were surveyed to detect HEV RNA in bile using reverse transcription quantitative polymerase chain reaction. The samples were then sequenced and genotyped.

Twenty pigs were positive for HEV ribonucleic acid, and seven samples had Ct values of less than 30. Among these 20 pigs, virus strains from 14 pigs were determined as genotype 3. This report indicated that HEV-contaminated pork liver was shipped to consumer markets and demonstrated the importance of detection of HEV in meat ready for shipment.

## 1. Introduction

**Figure 1.**
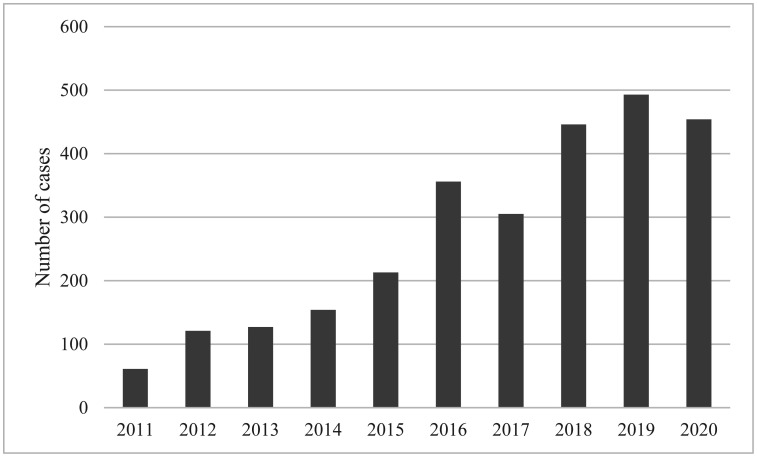
Annual numbers of confirmed hepatitis E cases in Japan between 2011 and 2020. Data available from Infectious Diseases Weekly Report (https://www.niid.go.jp/niid/ja/ydata/10411-report-ja2020-20.html) released from National Institute of Infectious Diseases. For 2020, the data were aggregated until October 30.

Hepatitis E is caused by hepatitis E virus (HEV) infection. In Europe, the number of confirmed cases increased tenfold from approximately 500 to >5,000 between 2005 and 2015, and 80% of these cases were detected in Germany, France, and the UK [1]. Izopet et al. reported that this increase was observed in most European countries and that the levels of HEV endemicity in countries, such as the Netherlands, France, Switzerland, and Germany are similar [2]. Germany has maintained a much greater pace of increase from 2016–2020 than the increase in reported cases prior to 2015, with >3,000 cases reported annually [3]. In Japan, hepatitis E is under surveillance and mandatory reporting, although the number of confirmed cases per population is very small compared to that in Germany; however, the number of confirmed hepatitis E cases is increasing (Figure 1) [4]. Of note, most of these cases are domestic transmission cases [5]. According to studies on blood donors in Japan, increasing trends of HEV RNA-positive donors were reported in Hokkaido between 2005 and 2019 [6], and national HEV RNA screening in Japan among blood donors has been ongoing since August 2020, with a reported positive rate of 0.055% [5].

The major route of HEV infection in humans is through consumption of undercooked pork-related foods [1],[3],[7]. HEV seroprevalence is high in farm pigs [8]–[12], and HEV has been detected in pork-related foods [13]–[18]. Although there are no reports of direct involvement of pork products in food-borne HEV infections in Japan, Yazaki et al. detected HEV RNA in liver from retail products in 2003 [19], and consumption of pork-related foods accounted for 41% of the cases reported in an outbreak investigation [5]. As in European countries, the involvement of pork-related foods in food-borne HEV infection is likely to be significant.

In Japan, HEV RNA prevalence was reported to be the highest in 3-month-old farm pigs, and HEV RNA was not detected in feces from 6-month-old pigs ready for shipment [20]. Motoya et al. reported that a serological survey of pigs at a farm in Ibaraki Prefecture showed that most pigs were infected with HEV during growth and were antibody-positive at the time of shipment [21]. Notably, Sasaki et al. reported that a survey of pig livers at one slaughterhouse in Miyagi Prefecture detected HEV RNA in >5% of shipped pigs [22].

There is limited HEV surveillance data in pigs and pork products in Japan, making it difficult to understand the actual status of HEV contamination of pork-related foods distributed in Japanese markets. In this report, we examined pig bile collected at a slaughterhouse to detect the presence of HEV RNA and investigate HEV infection among pigs slaughtered for food in Japan.

Since HEV multiplies in the liver and reaches the gastrointestinal tract while in bile, and HEV RNA can also be detected in feces, we focused on bile that accumulates in the gallbladder as a sample for the HEV RNA study. During a one-month period from February to March 2020, we collected gallbladders from 200 pigs shipped from 14 farms in seven Prefectures in eastern Japan for slaughter. HEV RNA was detected in 20 pigs slaughtered for shipment to markets.

## 2. Materials and methods

A total of 14 farms in seven Prefectures were sampled; farm 4 was sampled twice (Table 1). Either 10 or 20 Pigs per farm, or one or two farms were sampled in a single day. Therefore, among pigs slaughtered for human consumption at a slaughterhouse in the Kanto region between February and March 2020, gallbladders of 200 pigs were collected and delivered to the laboratory on the same day. One milliliter of bile in the gallbladder was collected and centrifuged at 3,000 × *g* for 10 min, and viral RNA was extracted from 200 µL of the supernatant after centrifugation using a magnetic bead extraction method (Maxwell RSC Viral Total Nucleic Acid purification kit; Promega, Madison, WI, USA).

**Table 1.** Bile sampling plan and RT-qPCR results.

Date	Farm	Prefecture	Number of samples	HEV RNA positive pigs	Ct value range
Feb 3	1	A	20		
Feb 3	2	B	20		
Feb 6	3	A	20		
Feb 12	4	B	20	2	38.2
Feb 13	5	B	20	4	21.2–28.6
Feb 25	6	F	10		
Feb 25	7	C	10	3	32.3–41.4
Feb 26	8	C	10		
Feb 26	9	E	10		
Feb 27	4	B	10		
Feb 27	10	D	10		
Feb 28	11	G	10		
Feb 28	12	D	10		
Mar 3	13	C	10	5	25.2–36.0
Mar 3	14	C	10	6	27.4–40.0
Total	14	7	200	20	21.2–41.4

One-step reverse transcription qualitative polymerase chain reaction (RT-qPCR) was used to detect HEV RNA using primer pairs JV-HEV-F; 5′-GGGTGGTTTCTGGGGTGAC-3′ and JV-HEV-R; 5′-AGGGGTTGGTTGGGATGAA-3′ and probe JV-HEV-P; 5′ 6FAM/TGATTCTCAGCCCTTCGC-3′-TAMRA [23]. Reaction tubes (RICOH standard DNA, RICOH, Tokyo, Japan) containing four copies of synthesized plasmid with the target region per tube, were used as a positive control for RT-qPCR. The synthesized plasmids were dispensed into each reaction tube using a bioprinting application [24]. Four copies/tube of HEV DNA were detected in duplicate each time. For RT-qPCR-positive samples, primer pairs HEV-F1; 5′-TAYCGHAAYCAAGGHTGGCG-3′ and HEV-R2; 5′-TGYTGGTTRTCRTARTCCTG-3′ and HEV-F2; 5′-GGBGTBGCNGAGGAGGAGGC-3′ and HEV-R1; 5′-CGACGAAATYAATTCTGTCG-3′ were used in the first and second rounds of nested PCR, respectively. This nested PCR was used to determine HEV genotypes based on the ORF2 capsid gene, nucleotide positions 5,963–6,340 (AF082843.1), reported by Tam et al. [25]. The amplified products were directly sequenced. The construction of phylogenetic tree and subtyping of HEV genotype 3 were performed according to current reference sequences [26] using GENETYX version 14 (GENETYX Software Development, Tokyo, Japan).

## 3. Results

Gallbladders were collected from pigs shipped from 14 farms from February 3 to March 3, 2020, on nine separate days. The numbers of samples per Prefecture were 40, 70, 40, 20, and 10 for Prefectures A; B; C; D; and E, F, and G, respectively.

**Figure 2.**
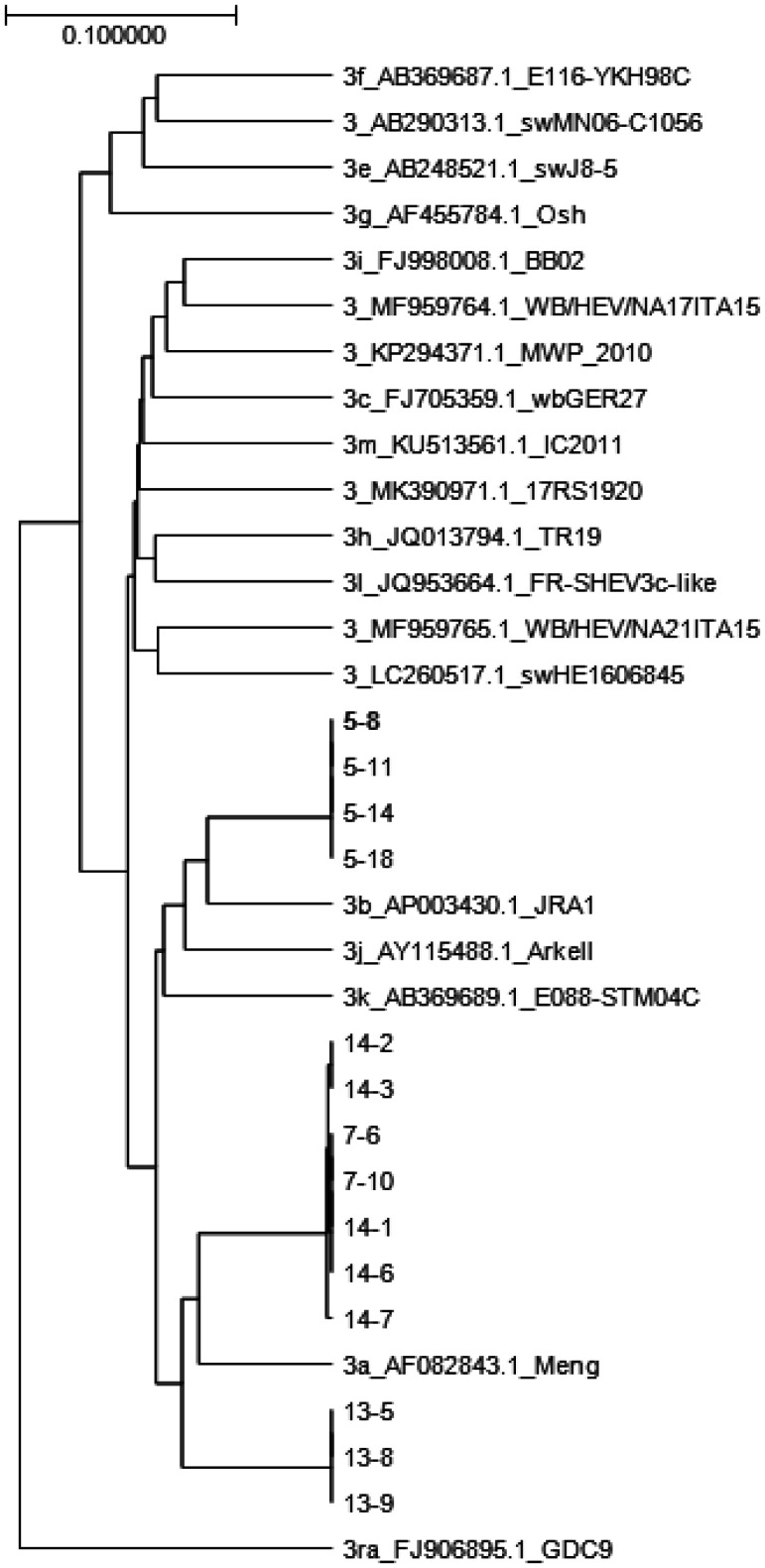
Genotype analysis of 14 virus strains was conducted using GENETYX ver14 software with the UPGMA method based on the Kimura's 2 parameter model. These strains belonged to genotype 3 and have been labeled in the phylogenetic tree as ‘farm number – pig number’.

A total of 20 pigs shipped from five (Farms 4, 5, 7, 13, and 14) farms were positive for HEV RNA. These five farms were distributed in two Prefectures (B and C), with a positivity rate of 8.6% (6/70) and 35% (14/40), respectively. Positive rates per farm were 6.7% (2/30), 20% (4/20), 30% (3/10), 50% (5/10), and 60% (6/10), for farms 4, 5, 7, 13 and 14, respectively. Of the 20 pigs that tested positive for HEV RNA, Ct values ranged from 21.2 to 41.4, with a mean of 32.3 and a median of 32.9. Seven pigs had Ct values <30, and 13 pigs had Ct values >30 (including 9 pigs with Ct values >35). The Ct values per farm were 38.2, 21.2–28.6, 32.3–41.4, 25.2–36.0, and 27.4–40.0 for farms 4, 5, 7, 13, and 14, respectively. Among the 20 HEV RNA-positive samples, partial sequences of the capsid gene could be determined for 14 and these were classified as genotype 3. Among these, 10 strains were subtyped as 3a, and 4 strains as 3b (Figure 2).

## 4. Discussion

HEV RNA was detected in 10% (20/200 pigs) of the pig bile samples, which is also consistent with the 2019 results from Sasaki et al. [22]. HEV RNA-positive pigs were shipped from five farms in two Prefectures. The HEV infection status may differ between farms, and since no serologic survey was conducted, we could not determine the history of HEV infection at the fattening stage. Since Motoya et al. reported that most pigs become infected with HEV during rearing in Ibaraki Prefecture in eastern Japan [21], it is likely that a similar situation occurred among the farms involved in this survey.

Among the 14 farms, pigs from farm 4 were sampled twice (February 12 and 27); the first sampling had a positivity rate of 10%, and the second was negative, suggesting that the HEV RNA positivity or HEV infection status may vary depending on the lot shipments, even when the pigs are shipped from the same farm. Further investigation, such as year-round HEV RNA monitoring of pigs in a farm, will help determine the HEV contamination status.

Of the 20 HEV RNA-positive samples, nested PCR products could be obtained from 14 samples. We determined the ORF2 capsid sequences in these samples and identified them by phylogenetic tree analysis. Ten samples belonged to subtype 3a, while 4 belonged to subtype 3b. This result is consistent with previous reports [5],[6],[20],[22] reporting that most HEV isolates belonged to subtype 3a or 3b in Japan.

Seven out of the 20 HEV RNA-positive pigs had a Ct value of <30, with a minimum value of 21.2, suggesting that some pigs may retain infectious HEV and shed the virus at the time of slaughter, thus, allowing highly HEV-contaminated pork to be distributed in the markets for human consumption. To estimate the risk of HEV infection via pork-related foods, further investigations are needed to determine the relationship between the level of HEV RNA in bile and HEV contamination of the liver and pork products.

García et al. reported high HEV RNA detection rate in liver and feces among specimens in a study at a slaughterhouse [17]. In general, it is necessary to sample several small portions of liver or meat for test. However, this may result in significant losses if whole livers are discarded based on this sampling method. On the other hand, livers can be distributed to consumer markets because cholecystectomy is less invasive to the liver. Of note, bile has fewer cellular components than liver fragments or feces; thus, pretreatment for RNA extraction is simpler, and its transportation is easier from the collection site to the laboratory. Therefore, bile collection is a suitable method for investigating HEV infection status in pigs at slaughterhouses.

This study had limitations. In Japan, most pigs are shipped at the age of 6 months. However, the actual shipment is determined by the farm according to the growth conditions, and pigs younger than 6 months can also be shipped. Hence, our data may be biased due to the unintentional exclusion of these younger shipment-ready pigs, which could have been infected. Secondly, our survey period was only a month, and the number of samples from the shipping area where HEV RNA was not detected was small. Hence, a longer survey period and a larger sample size would be required to understand the actual status of HEV infection among pigs at slaughterhouses.

## 5. Conclusions

Raw pork-related foods, such as pork sausage and liver paste, are not common in Japan, and the possibility of a large-scale increase in hepatitis E cases as reported in Germany is unlikely. However, our study indicated the potential distribution of HEV-contaminated pork liver and pork meat, and highlighted the importance of conducting epidemiological surveys and appropriate risk communication regarding hepatitis E to maintain the incidence of hepatitis E at low levels in Japan in the future.
